# Sustaining Rwanda's HIV response after elimination of PEPFAR funding: a modelling analysis of HIV epidemic and care continuum outcomes

**DOI:** 10.1002/jia2.70078

**Published:** 2026-02-11

**Authors:** April D. Kimmel, Zhongzhe Pan, Gad Murenzi, Ellen Brazier, Batya Elul, Benjamin Muhoza, Marcel Yotebieng, Kathryn Anastos, Denis Nash, and on behalf of the Central Africa International epidemiology Databases to Evaluate AIDS (CA‐IeDEA)

**Affiliations:** ^1^ Department of Health Policy Virginia Commonwealth University School of Public Health Richmond Virginia USA; ^2^ Division of Infectious Diseases Department of Internal Medicine Virginia Commonwealth University School of Medicine Richmond Virginia USA; ^3^ School of Public Health, College of Medicine and Health Sciences University of Rwanda Kigali Rwanda; ^4^ Institute for Implementation Science in Population Health City University of New York New York New York USA; ^5^ Columbia University Mailman School of Public Health New York New York USA; ^6^ Albert Einstein College of Medicine New York New York USA; ^7^ Department of Epidemiology and Biostatistics City University of New York Graduate School of Public Health New York New York USA

**Keywords:** care continuum, epidemic, funding, HIV and AIDS, mathematical model, Rwanda

## Abstract

**Introduction:**

HIV prevention and treatment supported by the United States President's Emergency Plan for AIDS Relief (PEPFAR) have saved millions of lives. Rwanda is among the most successful countries worldwide in achieving global targets with PEPFAR support. Abrupt PEPFAR funding uncertainty raises concerns about continued HIV epidemic control. We projected the impact of the Government of Rwanda's (GoR's) capacity to offset PEPFAR funding elimination on adult HIV epidemic and care continuum outcomes over 10 years.

**Methods:**

Using an HIV policy model calibrated to Rwanda, we assessed capacity to sustain HIV services at: 50% (with no capacity by GoR to cover the PEPFAR funding gap), 75%, 90% and 100% (with full capacity by GoR to cover the gap). Scenarios involved reducing the number on antiretroviral therapy (ART), immediately discontinuing ART and proportionally decreasing HIV diagnosis, ART initiation, and care re‐engagement. We projected epidemic outcomes (HIV prevalence, HIV incidence, number with HIV, new HIV infections, deaths) and care continuum outcomes (percentage diagnosed, percentage on ART among those diagnosed, percentage virally suppressed among those on ART). We calculated differences in projected outcomes for partial or no capacity versus full capacity. Secondary analyses assessed the timing of the GoR's response.

**Results:**

Compared to full capacity at 10 years, the model projected a 13.9%–38.7% increase in HIV prevalence and 69.0%–246.7% increase in HIV incidence across coverage capacity scenarios. This translated to 29,000–64,000 additional adults with HIV and 20,000–92,000 cumulative new adult HIV infections. Cumulative projected deaths increased by 10,000–51,200. The model projected continual reductions in percentage diagnosed at 10 years; percentage virally suppressed among those on ART was similar across scenarios. Higher, and more delayed, coverage capacity had projected outcomes similar to lower, and less delayed, coverage capacity. Outcomes for gradual increases in coverage capacity were generally similar to or better than full, but delayed, coverage capacity.

**Conclusions:**

Even in countries like Rwanda that have achieved epidemic control, abrupt and persistent elimination of PEPFAR funding could drastically reverse critical gains. Evidence quantifying the consequences of different capacities to sustain HIV services underscores the high stakes of rapid and sufficient action.

## 1 INTRODUCTION

Rwanda is a global HIV success story, one of only nine countries worldwide achieving the 95‐95‐95 UNAIDS targets for epidemic control [1]. Rwanda's success relies substantially on commitment from the Government of Rwanda (GoR) through high levels of political will, a multisectoral response and implementation of evidence‐based approaches for HIV prevention and treatment [2]. While Rwanda has been moving towards sustainability of its HIV response [3], like many sub‐Saharan African countries, success of Rwanda's national response has hinged on support from external donors, especially the United States President's Emergency Plan for AIDS Relief (PEPFAR), which funded nearly 40% of the total HIV response and 50% of antiretroviral therapy (ART) in Rwanda, fiscal year 2023–2024 [4, 5].

PEPFAR's future, however, remains uncertain. Programme reauthorization by the US Congress was for only 1 year (vs. prior 5‐year reauthorizations) until 25 March 2025 [6]. On 20 January 2025, President Trump froze all US foreign aid for 90 days [7], although PEPFAR secured a limited waiver allowing some service continuation [8]. An abrupt, persistent elimination of PEPFAR funding will result in an approximately $60‐million‐dollar funding gap for Rwanda's national response and setbacks in epidemic control, despite the GoR's strong willingness to increase domestic HIV funding and anticipated temporary stocks of HIV treatment and laboratory testing supplies [9, 10, 11].

Leveraging the cohort study of the Central Africa International epidemiology Database on AIDS (CA‐IeDEA) and the calibrated CA‐IeDEA Rwanda HIV policy model [12], we projected the impact of the capacity for sustained HIV services, including the GoR's coverage of the PEPFAR funding gap, on adult HIV epidemic and care continuum outcomes.

## 2 METHODS

This study was approved by the Republic of Rwanda National Ethics Committee (RNEC660), Albert Einstein College of Medicine Office of Human Affairs (2021‐13394) and Virginia Commonwealth University Institutional Review Board (HM20008203). Consent was not obtained, as the study used secondary data with no identifiable information. Data were collected between 3 October 2024, and 28 February 2025.

### 2.1 Scenarios

2.1

Scenarios included: *50% sustained* (no capacity by the GoR to sustain HIV services and cover the PEPFAR funding gap), *75% sustained*, *90% sustained* and *100% sustained* (full capacity). Scenarios assumed complete, persistent and immediate elimination of PEPFAR support over 10 years starting in 2025 [4, 5]. Scenarios were operationalized by reducing the total on ART [13]. *50% sustained* assumed a 50% reduction in the number on ART, *75% sustained* a 25% reduction and *90% sustained* a 10% reduction. *100% sustained* assumed no reduction in the number on ART or no reduction in PEPFAR funding. Scenarios were constant over time.

In secondary analysis, we examined the timing of Rwanda's national response. We assessed the base case scenarios assuming 1‐ and 3‐year temporary delays in response. We also projected the impact of gradual increases in the GoR's response: (1) *slow* (50% sustained in year 1, 75% in year 2, 90% in year 3, 100% in years 4+); (2) *moderate* (75% sustained in year 1, 90% in year 2, 100% in years 3+); and (3) *rapid* (90% sustained in year 1, 100% in years 2+).

### 2.2 Model

2.2

We used the CA‐IeDEA Rwanda HIV policy model, a deterministic dynamic model of the HIV epidemic among adults aged 15–64 years [12]. The model includes compartments for HIV disease progression by CD4 stratum and care engagement and has 35 sub‐populations by age, sex, risk and urbanicity. Calibrated to Rwanda [12], the model projects epidemic and care continuum outcomes. Epidemic outcomes include: HIV prevalence (%), HIV incidence (number of new infections per 1000 population), annual number of new HIV infections, number with HIV and cumulative HIV‐related deaths. Care continuum outcomes include the percentage: diagnosed with HIV, on ART among those diagnosed, HIV virally suppressed among those on ART and on ART among those with HIV. We reported incremental differences and percentage change in mean projected outcomes for *50% sustained*, *75% sustained* and *25% sustained* compared, separately, to *100% sustained* between 2025 and 2035.

### 2.3 Model inputs and calibration

2.3

Model inputs were derived from individual‐level longitudinal data from the Rwanda cohort of CA‐IeDEA [14], estimated from Rwanda national surveys and population projections [15, 16, 17, 18, 19, 20, 21], and extracted from literature specific to Rwanda [9, 22, 23, 24, 25, 26, 27, 28, 29, 30, 31, 32, 33] (Table 1). The model calibration process is described elsewhere [12] and summarized in the Supplement; calibrated parameter values are in Table 1.

**Table 1:** Select model parameters

Parameter	Baseline value(s)^a^	Original data source(s)^a^			
Force of infection^b^					
Probability of HIV transmission per unprotected sexual act when not virally suppressed, by sub‐population^c^	0.0007–0.0108	Boily (2009) [24], Vittinghoff (1999) [25]			
Proportion consistently using condoms, by sub‐population^d^					
Lower‐risk women	0.032–0.224	DHS Rwanda (2005, 2010, 2015) [15, 16, 17]			
Lower‐risk men	0.108–0.560	DHS Rwanda (2005, 2010, 2015) [15, 16, 17]			
Female sex workers	0.275–0.353	BBSS (2010) [18, 19]			
Men who have sex with men	0.402–0.408	BBSS (2020) [18, 19]			
Average number of sexual acts per month, by sub‐population^e^					
Lower‐risk women and men	0.948–1.834	Braunstein (2011a) [28]			
Female sex workers	130.0	Braunstein (2011a) [28]			
Men who have sex with men	8.938	Binagwaho (2010) [29]			
Transition probabilities (monthly) for infectious compartments					
Susceptible population growth rates, by age, sex and urbanicity	0.08%–0.48%	World Bank [20]			
Natural history disease progression, by sex	0.033–0.049	CA‐IeDEA (Rwanda, 2004–2020) [14]			
Diagnosis, by CD4 stratum and year^f^	0.018–0.102	DHS Rwanda (2005, 2010, 2015) [15, 16, 17], Ntale (2019) [32], Okal (2017) [33]			
Linkage to care, by CD4 stratum	0.132–0.192	Braunstein (2011b) [30], Nsanzimana (2015) [31], Okal (2017) [33]			
Loss to follow‐up, by CD4 stratum and ART status	0.001–0.003	CA‐IeDEA (Rwanda, 2004–2020) [14]			
On ART and virally suppressed, by CD4 stratum and year^g^	0.129–0.265	CA‐IeDEA (Rwanda, 2004–2020) [14]			
On ART and virologic failure of available regimens, by CD4 stratum and year^g^	0.001–0.002	CA‐IeDEA (Rwanda, 2004–2020) [14]			
Re‐engagement in care^h^	0.022–0.024	CA‐IeDEA (Rwanda, 2004–2020) [14]			
Death on ART and virally suppressed, by sub‐population and CD4 stratum^i^	0.003–0.035	CA‐IeDEA (Rwanda, 2004–2020) [14]			
Calibration targets, most recent year					
HIV prevalence (15–64 years), overall and by sex	2.2%–3.7%	RPHIA (2019) [39]			
% on ART among diagnosed (15–44 years), overall and by sex	96.2%–97.2%	RPHIA (2019) [52]			
% virally suppressed among on ART (15–44 years), overall and by sex	83.1%–92.6%	RPHIA (2019) [52]			
Multiplicative adjustments for probabilities of diagnosis, ART initiation and ART re‐engagement					
	2025 adjustments^j^				2026+ adjustments^j^
	CD4 >500	CD4 >350–500	CD4 >200–350	CD4 <200	
100% sustained scenario	1	1	1	1	1
90% sustained scenario	0	0.4	0.6	0.9	1
75% sustained scenario	0	0.1	0.25	0.5	0.7
50% sustained scenario	0	0	0.05	0.1	0.3

Abbreviations: BBSS, Behavioral and Biological Surveillance Survey; CA‐IeDEA, Central Africa International epidemiology Databases to Evaluate AIDS; DHS, Demographic and Health Survey; RPHIA, Rwanda Population‐Based HIV Impact Assessment.

^a^ Model inputs were informed by both the data sources shown and the formal calibration process described in the Methods and previously [12]. The reported ranges reflect the mean of the top 50 best‐fitting parameter sets across specific sub‐populations and are used over the analytic time horizon of the current analysis. For context, we provide the original data sources from which the calibrated values were derived.

^b^ The force of infection reflects the probability of HIV acquisition, including male‐to‐female, female‐to‐male and male‐to‐male HIV transmission.

^c^ HIV transmission probability for male‐to‐female and female‐to‐male sexual contact captures both vaginal and anal sex. HIV transmission probability for male‐to‐male sexual contact reflects anal sex.

^d^ The range in the proportion consistently using condoms reflects differences across sub‐populations for a given projection period as well as within sub‐populations over time.

^e^ The average number of sexual acts per month varies by sub‐population.

^f^ The monthly probability of diagnosis varies across and within sub‐populations over time. The reported range is for 2025 only.

^g^ The monthly probability varies across and within sub‐populations over time. The reported range is for 2025 only.

^h^ Represents the baseline value for re‐engagement in care among those with CD4 cell count <200 cells/µl, which is adjusted by CD4 stratum.

^i^ The range in the monthly probability of death for those on ART and virally suppressed reflects differences across sub‐population and CD4 stratum immediately preceding ART initiation.

^j^ Adjustments were applied annually to monthly probabilities.

### 2.4 Model input adjustments to capture PEPFAR funding elimination

2.4

We adjusted the 2025 projected population distribution across compartments. Reductions in ART coverage were scenario‐specific and immediate and were applied proportionally to sub‐populations according to their distribution across compartments in 2025. Among those discontinuing ART in 2025, we assumed half remained in care with non‐ART‐related clinical support, while the remainder were lost from care. We assumed people virally suppressed, but for whom ART was abruptly discontinued due to PEPFAR funding elimination, had CD4 counts >500 cells/µl [34]; we also assumed those on ART and *not* virally suppressed, but for whom ART was abruptly discontinued, had CD4 counts >200–350 cells/µl [35].

We assumed changes in coverage capacity decreased probabilities of HIV diagnosis, ART initiation and re‐engagement in care [36]. Decreases reflect clinical and resource allocation decisions made due to limited availability of diagnostics, medication and related infrastructure, including clinic staffing, which can impact service delivery for HIV testing, ART, and retention and re‐engagement in care [36, 37, 38]; they also reflect behavioural changes resulting in delayed initial presentation to clinical care [36]. We modelled the decreases by applying multiplicative adjustments to parameters for HIV diagnosis, ART initiation and re‐engagement, assuming loss to follow‐up related to insufficient ART supply occurred immediately after funding cuts and was captured by sub‐population re‐distribution. Adjustments resulted in constant, scenario‐specific numbers on ART over time.

## 3 RESULTS

### 3.1 Baseline analysis

3.1

**Figure 1:**
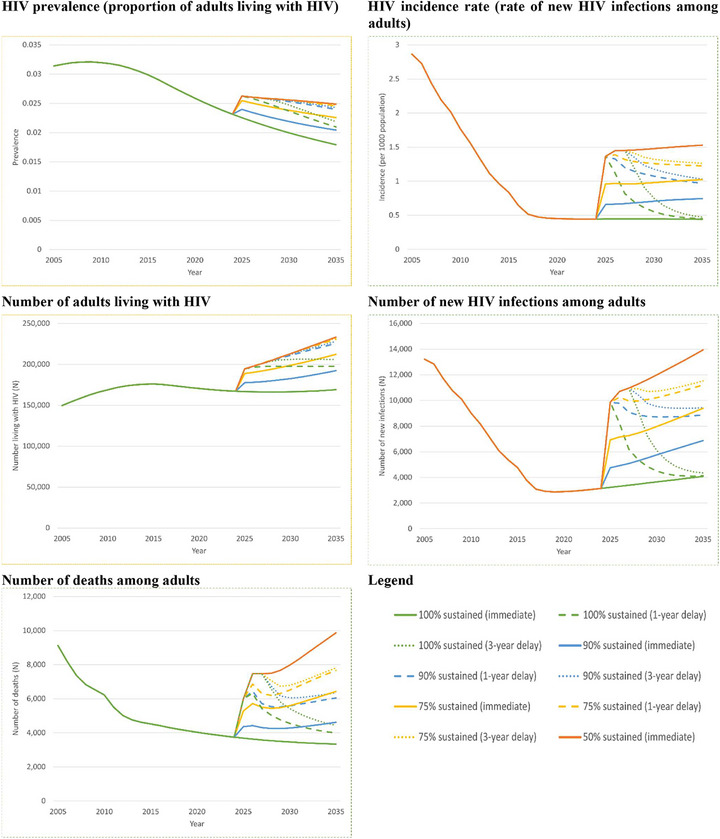
Projected HIV epidemic outcomes in Rwanda among adults aged 15–64 years by 2035.

Decreased capacity for the GoR to cover a complete, persistent PEPFAR funding gap worsened adult HIV epidemic outcomes (Figure 1). Compared to *100% sustained* at 10 years, HIV prevalence increased from 1.8% to 2.0% (*90% sustained*, +13.9%) and 2.5% (*50% sustained*, +38.7%). HIV incidence per 1000 population increased from 0.44 to 0.75 (*90% sustained*, +69.0%) and 1.53 (*50% sustained*, +246.7%). New HIV cases increased by nearly 2800 (+68.3%) and 9900 (+241.9%), respectively, in year 10, versus *100% sustained* in year 10. Ten‐year cumulative new HIV cases increased from 40, 000 to 63, 000 (*90% sustained*, +58.1%) and 132, 000 (*50% sustained*, +229.1%). Over 10 years, the number with HIV increased to 192, 000 (*90% sustained*, +13.8%) and 233, 000 (*50% sustained*, +38.0%). Ten‐year cumulative deaths increased to 48, 000 (*90% sustained*, +25.9%) and 90, 000 (*50% sustained*, +133.8%), versus 38, 000 deaths (*100% sustained*).

**Figure 2:**
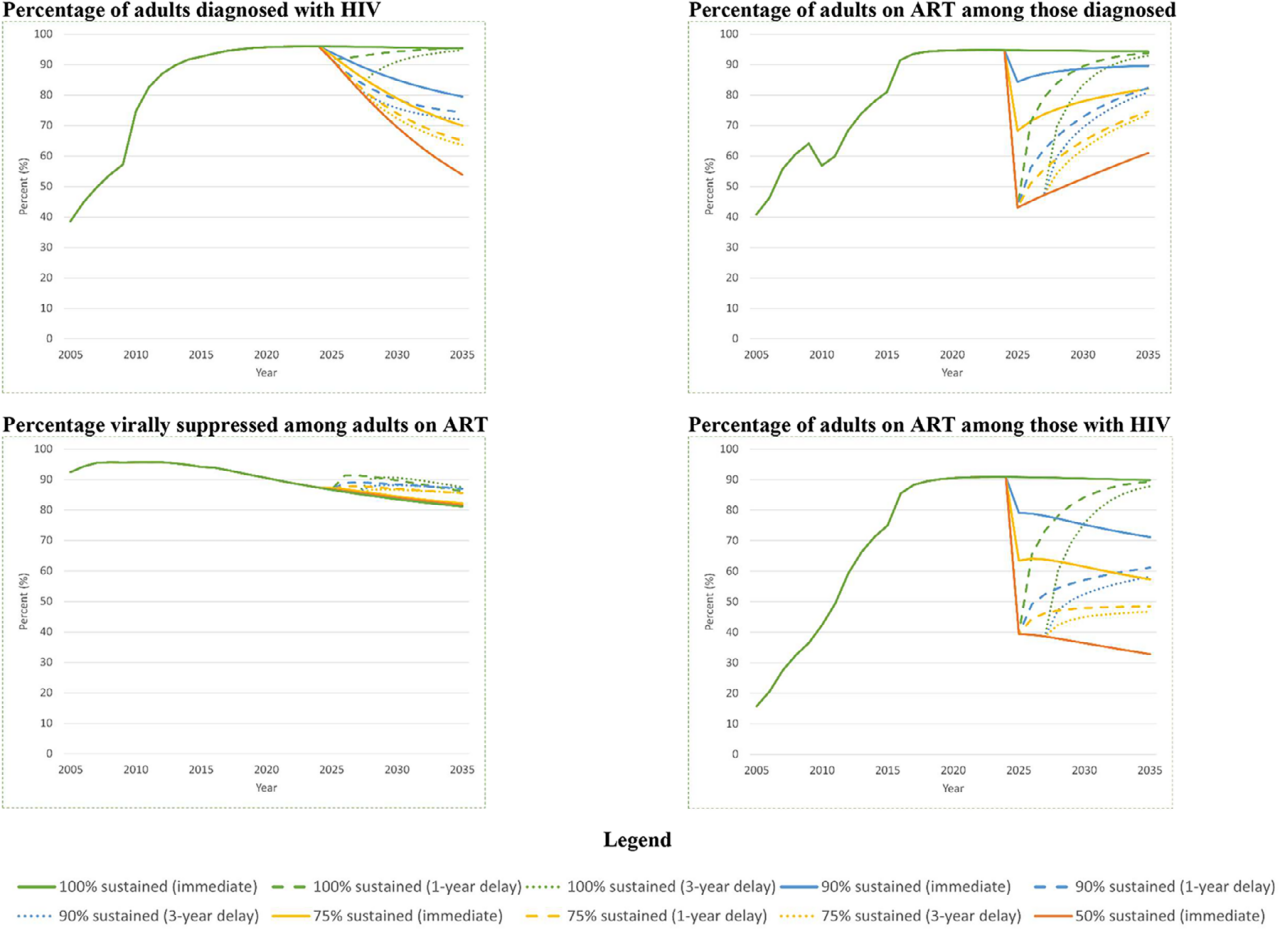
Projected HIV care continuum outcomes in Rwanda among adults aged 15–64 years by 2035.

Decreasing capacity by the GoR to cover a complete, persistent PEPFAR funding gap had a varied impact on HIV care continuum outcomes (Figure 2). The percentage diagnosed with HIV had continual reductions, with decreases from 95.3% for *100% sustained* to 79.5% (*90% sustained*, –16.6%) and 53.9% (*50% sustained*, –43.5%) by 2035. Similarly, the percentage on ART among those with HIV decreased from 90.0% for *100% sustained* to 71.2% (*90% sustained*, –20.7%) and 32.8% (*50% sustained*, –63.4%) in 10 years. The percentage on ART among diagnosed in 2025 had as high as a 54.5% decrease (*50% sustained* vs. *100% sustained*). However, these reductions diminished over time: in 2035, ART coverage among those diagnosed with HIV decreased from 94.3% for *100% sustained* to 89.6% (*90% sustained*, –5.0%) and 61.0% (*50% sustained*, –35.3%). Finally, the percentage virally suppressed given ART remained similar across scenarios (∼1% difference), but had a downward trend.

### 3.2 Secondary analysis

3.2

HIV epidemic outcomes were sensitive to the timing of the GoR's response. Outcomes generally worsened as delays in covering the PEPFAR funding gap grew, although the impact of the delays diminished as coverage capacity decreased. Higher, and more delayed, coverage capacity had outcomes similar to lower, and less delayed, coverage capacity. For example, by 2035, the number of new infections projected for a 3‐year delay in *90% sustained* was similar to immediate *75% sustained* (both 9400), while the number with HIV projected from a 3‐year delay in *75% sustained* was similar to *50% sustained* (232, 000 vs. 233, 000). When coverage capacity increased gradually, outcomes generally were similar to or better than for delayed full coverage. For example, the number with HIV for *slow* was similar to a 3‐year delay in *100% sustained* (205, 000 vs. 206, 000), while there were 5000–17, 000 fewer with HIV for *moderate* and *rapid* than for a 1‐year delay in *100% sustained*. At times, outcomes for *slow* were worse than for a 1‐year delay in *100% sustained*, such as for cumulative new infections (+23% increase).

For HIV care continuum outcomes, findings varied regarding the timing of the GoR's response. Higher, and more delayed, coverage capacity had outcomes similar to lower, and less delayed, coverage capacity. For example, by 2035, the percentage diagnosed for a 3‐year delay in *90% sustained* was similar to immediate *75% sustained* (72.0% vs. 69.9%), while the percentage on ART among diagnosed for a 1‐year delay in *90% sustained* was similar to immediate *75% sustained* (82.3% vs. 82.0%). Notably, delays in full coverage (*100% sustained*) had minimal impact on percentage diagnosed and percentage on ART among diagnosed versus immediate full coverage. However, across all scenarios, the percentage virally suppressed increased with coverage delays. Here, the percentage increase was highest for a 1‐year delay, although this generally decreased as coverage capacity diminished. Outcomes for gradual increases in coverage capacity generally were similar to those for delayed full coverage, although the percentage virally suppressed given ART worsened as the speed of response accelerated.

## 4 DISCUSSION

Given an abrupt, complete and persistent 10‐year withdrawal of PEPFAR funding, the decreasing capacity of the GoR to sustain PEPFAR‐supported HIV services worsened epidemic outcomes but had a varied impact on care continuum outcomes. Delayed coverage of the same coverage capacity worsened all outcomes except for full capacity, for which there was a minimal impact on some HIV epidemic and care continuum outcomes. Higher, and more delayed, coverage capacity had outcomes similar to lower, and less delayed, coverage capacity. HIV epidemic and care continuum outcomes for gradual increases in coverage capacity were generally similar to or better than for full, but delayed, coverage capacity.

Progressively lower capacity to cover the PEPAR funding gap substantially worsened HIV epidemic outcomes, but had a varied impact on care continuum outcomes. In particular, for HIV care continuum outcomes, there were immediate, sharp reductions in the percentage on ART among diagnosed, with these reductions diminishing over time, while the percentage virally suppressed given ART remained similar, although gradually decreasing, across all coverage capacities. The former is due to reductions in the number diagnosed with HIV and by stable, scenario‐specific numbers on ART over time. That is, for people diagnosed with HIV who have either discontinued ART or cannot initiate ART due to resource constraints, downstream consequences of lack of treatment may include virologic failure, disease progression and higher risk of AIDS‐related illness, which can lead to increased mortality. Increased mortality, combined with the stable, scenario‐specific numbers on ART, can reduce the number diagnosed with HIV and thus, increase the percentage on ART among diagnosed. The latter is due to our assumption that provision of treatment and care for those on ART was similar regardless of capacity constraints [39]. The downward trend in the percentage virally suppressed given ART across all scenarios reflects the increasing percentage on ART having virologically failed all available ART regimens, highlighting the importance of making available multiple therapeutic drug classes for the treatment of HIV to continue to achieve targets even when there are capacity constraints.

Delayed capacity to cover the PEPFAR funding gap worsened HIV epidemic and care continuum outcomes, except for the full coverage capacity scenario and one outcome: percentage virally suppressed given ART. Notably, HIV prevalence in the presence of a delay was similar regardless of coverage capacity, except for full capacity. This is due to the immediate, sharp increase in new infections, which could have long‐lasting effects on HIV prevalence and number with HIV. Surprisingly, the percentage virally suppressed given ART increased with coverage capacity delays. This is because, compared to no delays, a greater number for whom ART was discontinued progressed to lower CD4 cell counts. In our model, those with lower CD4 cell counts are more likely to initiate ART than those with higher CD4 cell counts; therefore, a greater number initiated ART and became virally suppressed, resulting in a higher proportion virally suppressed than without coverage delays.

When there were gradual increases in coverage capacity, HIV epidemic and care continuum outcomes were generally similar to or better than for full, but delayed, coverage. At times, compared to shorter delays in full coverage, outcomes worsened for slow increases (e.g. for cumulative new HIV infections) and moderate to rapid increases (e.g. for percentage virally suppressed given ART) in coverage capacity. The former is due to a longer time with insufficient ART supply, which increased the percentage virally suppressed. The latter can be explained by the more rapid consumption of all available ART regimens due to faster recovery of the ART supply.

Findings suggest that a swift response to the PEPFAR funding gap, even if with limited or gradual increases in coverage capacity, could have similar or better outcomes than delayed but higher coverage capacity. The GoR is intensifying its efforts to self‐sustain HIV programmes and limit the impact of the abrupt HIV financing gap left by PEPFAR, which information suggests has disrupted availability of HIV test kit, viral load test kit, and ART and has interrupted service delivery due to staffing layoffs or clinic closures [11, 40]. In response, the GoR is increasing government budgets alongside economic development [41], as well as engaging the private sector to sustain the HIV response [4, 42]. With continued effort and political priority of self‐sustaining HIV programmes, Rwanda has the potential to begin covering the PEPFAR funding gap with limited delay in initiating this coverage. Doing so may provide important insights for other countries facing similar situations and mobilizing to respond. Systematically collecting data on site‐ and programme‐level evolution alongside the GoR's sustainment efforts could, additionally, substantially improve situational awareness and future modelling efforts. However, such data collection may be challenging in the current context, given pressing priorities imposed by the funding crisis.

The HIV policy model used in this study was developed and calibrated using the Rwanda cohort of CA‐IeDEA. Cohort collaborations, such as CA‐IeDEA, have played an important role in guiding Rwanda's HIV response and across the sub‐Saharan Africa region. These cohorts provide the real‐world, longitudinal data necessary for Ministries of Health to evaluate programme impact, identify areas where course corrections may be needed and plan for sustainability. Leveraging these data to inform policy underscores the importance of cohort‐based infrastructure in supporting national decision‐making over the coming months and years. Their use in the current study highlights the importance of supporting Ministries of Health in using robust analytic infrastructure—including cohort data platforms—to inform programmatic planning and mitigate the impacts of abrupt transitions to government health programmes.

Our study is among the first to project the impact of sustaining HIV programmes on HIV epidemic and care continuum outcomes, given the elimination of PEPFAR funding. Our findings support that abrupt elimination of PEPFAR funding could have a significant impact on progress towards ending the HIV epidemic as a public health threat, which had largely been achieved by the GoR, with 50% of its total ART budget supported by PEPFAR [5]. Our findings align with other modelling studies done in low‐ and middle‐income countries (LMICs). In South Africa, where PEPFAR funding supported 11% of the total HIV budget, a full extraction of PEPFAR funds resulted in a 50% increase in HIV incidence in 10 years and reduced the percentage diagnosed and percentage on ART among diagnosed by 3% and 14% in 5 years, respectively [43]. Among 26 LMICs where PEPFAR supports 45% of total HIV spending, a full extraction of PEPFAR funds resulted in a 283% increase in cumulative new HIV infections and 191% increase in HIV‐related deaths over 5 years [44]. These align with our finding that sustaining Rwanda's HIV budget could result in a 70%–247% increase in HIV incidence, 58%–229% increase in cumulative new infections, 17%–44% reduction in percentage diagnosed and 5%–35% reduction in percentage on ART among diagnosed. The differences in magnitude are possibly because our scenarios were defined by only restricting ART availability, which other work assumed was not affected or affected additional HIV‐related programmes (e.g. prevention programmes) besides ART. Further, our findings of increased cumulative deaths (1460–5120 annually) are consistent with the preliminary assessment of excess HIV‐related deaths (562 in 1 year) due to pauses in PEPFAR funding [45]. Our projected cumulative deaths are likely higher because we examined a persistent elimination of PEPFAR funding versus a 90‐day pause in funding.

Our study has limitations. First, we assumed immediate, scenario‐specific discontinuation of ART, although reports suggest ART stockouts were not expected for 3–6 months [11]. Therefore, we may have overestimated the impact of PEPFAR funding elimination. Conversely, in defining scenarios in terms of ART coverage decreases only, we did not capture disruptions in staff availability to deliver ART and related services. While we operationalized our scenarios to align with survey findings on the ability to deliver HIV services—including the frequent cancellations and reduced activity related to HIV testing, HIV treatment and services for care re‐engagement [36]—after the extraction of PEPFAR funding, our approach to capture staff shortages may have underestimated the impact of PEPFAR funding elimination on outcomes. Second, we examined the impact of PEPFAR funding elimination only on individuals aged 15–64 years and did not assess its impact on HIV prevention services (e.g. pre‐exposure prophylaxis (PrEP)) and mother‐to‐child transmission. This is because our model structure does not allow for this assessment; therefore, model projections do not reflect the potential impact on infants, children and adolescents at risk for and living with HIV. Thus, our projections are limited in terms of these groups and by definition do not estimate the overall impact on epidemiologic and care continuum outcomes of PEPFAR funding elimination. Third, we assumed that when the PEPFAR funding gap was covered, HIV testing and ART would re‐initiate at a rate similar to the PEPFAR funding elimination. However, the extraction of PEPFAR funding may result in health workforce layoffs and closure of HIV‐related infrastructure [46], which could have a long‐lasting impact on HIV service delivery and population outcomes. Fourth, given the lack of data, we assumed scenario‐specific reductions in ART coverage were applied proportionally to sub‐populations according to their 2025 distribution across compartments, although this assumption can be updated as new data emerge. Fifth, model simplifications may have resulted in inexact model projections, particularly for key populations. We neither modelled acute HIV nor sexual contact between MSM and heterosexual populations. We did not parameterize the model to reflect trends in condom use among female sex workers (FSWs) and men who have sex with men (MSM), as the condom use time horizon (i.e. most recent sex act, in the past month, in the past 12 months) was reported inconsistently, and because we did not model partner types. For example, for MSM, data on consistent condom use in the past month were available in later reports [47, 48], but not in earlier reports [49]. For FSWs, while data on consistent condom use in the past month were available over time, the data were not consistent in terms of partner type—reported overall only [18, 49] or disaggregated by partner type only [47, 48]—with no information on partner type distribution. We also did not calibrate the model to historical data on FSWs and MSM, although some literature suggests lower antiretroviral coverage and viral suppression in these sub‐populations [23, 50, 51] versus the overall population [52]. Ultimately, our parameterization and calibration simplifications for FSWs and MSM may have resulted in overestimates of analysis outcomes. Future research projecting the impact of PEPFAR elimination, particularly for key populations, will benefit from model updates that address these simplifications. Finally, we did not explicitly model the death of the susceptible population. Rather, we modelled the average rates of population growth in susceptible compartments by sub‐population, an approach that simultaneously captures entry, maturation and mortality within each compartment. We implemented this approach given its mathematical tractability, coupled with the availability of historical data on the size of the adult population in Rwanda, for multiple sub‐populations, over time [53]. However, using a fixed population growth rate for a given sub‐population, as in our model, may result in artificially lower birth rates or may eclipse short‐term variation or decreasing trends in population growth that a model structure with separate parameters related to birth, death and maturation rates may otherwise capture. Nonetheless, prior work suggests that model projections fit multiple sources of historical data and can be used to inform population policy in Rwanda [12].

## 5 CONCLUSIONS

The ability to sustain HIV budgets, given the persistent elimination of PEPFAR funding, would greatly limit progress to end the public health threat posed by the HIV epidemic and the achievement of 95‐95‐95 UNAIDS targets in Rwanda. Rapid response to cover the PEPFAR funding gap and to sustain HIV treatment and prevention programmes is key to minimizing the effect of PEPFAR funding elimination in Rwanda.

## COMPETING INTERESTS

The authors declare no competing interests.

## AUTHOR CONTRIBUTIONS

ADK and GM conceptualized the research. ADK and ZP designed the analysis. ZP conducted the analysis. ADK and ZP drafted the manuscript. ADK, ZP, GM, EB, BE, BM, MY, KA and DN interpreted the data and critically reviewed and revised the manuscript.

## FUNDING

Research reported in this publication was supported by the U.S. National Institutes of Health's (NIH) National Institute of Allergy and Infectious Diseases, the *Eunice Kennedy Shriver* National Institute of Child Health and Human Development, the National Cancer Institute, the National Institute of Mental Health, the National Institute on Drug Abuse, the National Heart, Lung, and Blood Institute, the National Institute on Alcohol Abuse and Alcoholism, the National Institute of Diabetes and Digestive and Kidney Diseases and the Fogarty International Center under Award Number U01AI096299 (Central Africa IeDEA). Informatics resources are supported by the Harmonist project under Award Number, R24AI24872.

## DISCLAIMER

This work is solely the responsibility of the authors and does not necessarily represent the official views of any of the institutions mentioned above. This work is subject to the NIH Public Access Policy. Through acceptance of this federal funding, NIH has been given a right to make this manuscript publicly available in PubMed Central upon the Official Date of Publication, as defined by NIH.

## Supporting information




Supporting Information

## Data Availability

The data used to populate the policy model and that support the findings of this study are available in Table 1, as well as in the main text and supplementary content of: Kimmel AD, Pan Z, Brazier E, Murenzi G, Mujwara D, Muhoza B, Yotebieng M, Anastos K, Nash D; Central Africa International epidemiology Databases to Evaluate AIDS (CA‐IeDEA). Development and calibration of a mathematical model of HIV outcomes among Rwandan adults: Informing achievement of global targets across sub‐populations in Rwanda. *PLoS One* 2025;20(5): e0310662. https://doi.org/10.1371/journal.pone.0310662.

## References

[1] Joint United Nations Programme on HIV/AIDS . AIDSinfo: Global data on HIV epidimiology and response. 2024. [cited 2025 March 21]. Available from: https://aidsinfo.unaids.org/.

[2] Rwanda Ministry of Health . National HIV/AIDS targets: 2018‐2020‐2030. 2015.

[3] Joint United Nations Programme on HIV/AIDS . HIV sustainability planning: analytical resource. 2024 [cited 2025 March 21]. Available from: https://sustainability.unaids.org/wp‐content/uploads/2024/08/Rwanda__‐Executive‐Summary‐May‐2024.pdf.

[4] Joint United Nations Programme on HIV/AIDS . Impact of US funding cuts on HIV programmes in Rwanda. 2025. accessed January 21, 2026. Available from: https://www.unaids.org/en/resources/presscentre/featurestories/2025/march/20250318_Rwanda_fs.

[5] Centers for Disease Control and Prevention Global Health Center . CDC in Rwanda: HIV and TB. 2024. [cited 2025 April 15]. Available from: https://www.cdc.gov/global‐health/countries/rwanda.html#cdc_generic_section_3‐hiv‐and‐tb.

[6] Consolidated Appropriations Act, 2024, Publ. L. No. 118‐42, 138 Stat. 460 (2024). Accessed January 21, 2026. https://www.congress.gov/bill/118th‐congress/house‐bill/2882/text..

[7] The White House . Executive Order, Reevaluating and Realigning United States Foreign Aid. 2025. [updated January 20, 2025]. Available from: https://www.whitehouse.gov/presidential‐actions/2025/01/reevaluating‐and‐realigning‐united‐states‐foreign‐aid/.

[8] United States Department of State . Info Memo for the PEPFAR Implementing Agencies and PEPFAR Country Coordinators. 2025. [updated February 1, 2025]. Available from: https://docs.google.com/document/u/0/d/e/2PACX‐1vQgNTpC6F5oaOnkebQokJ_2eYgM_IcQNT7alIL6R3P16Ef4Z0pmQby3Y1eHbJcTxK_yJ8EPVNiibxON/pub?pli=1.

[9] The U.S. President's Emergency Plan for AIDS Relief . Rwanda country operational plan (COP/ROP) 2023 strategic direction summary. 2022. accessed January 21, 2026. Available from: https://www.prepwatch.org/wp‐content/uploads/2024/06/Rwanda‐Strategic‐Direction‐Summary‐2023.pdf..

[10] United States Department of State . Fiscal Years (FY) 2024 and 2025 PEPFAR Planned Allocation. 2023. [cited 2025 April 15]. Available from: https://www.state.gov/wp‐content/uploads/2023/02/Rwanda‐COP23‐PLL_02_15_2023.pdf.

[11] Joint United Nations Programme on HIV/AIDS . Impact of US funding cuts on HIV programmes in Rwanda. 2025. [cited 2025 March 18]. Available from: https://www.unaids.org/en/resources/presscentre/featurestories/2025/march/20250318_Rwanda_fs.

[12] Kimmel AD , Pan Z , Brazier E , Murenzi G , Mujwara D , Muhoza B , et al. Development and calibration of a mathematical model of HIV outcomes among Rwandan adults: informing achievement of global targets across sub‐populations in Rwanda. PLoS One. 2025;20(5):e0310662. 40367028 10.1371/journal.pone.0310662 PMC12077668

[13] Kimmel AD , Charles M , Deschamps M‐M , Severe P , Edwards AM , Johnson WD , et al. Lives saved by expanding HIV treatment availability in resource‐limited settings: the example of Haiti. J Acquir Immune Defic Syndr. 2013;63(2):e40–e48. 23535289 10.1097/QAI.0b013e3182918875 PMC3821389

[14] National Institute of Allergy and Infectious Diseases . Central Africa International epidemiology Databases to Evaluate AIDS. National Institute of Allergy and Infectious Diseases. accessed January 21, 2026. Available from: https://ca‐iedea.org/.

[15] Institut National de la Statistique (INSR) [Rwanda], ORC Macro . Rwanda Demographic and Health Survey 2005. Calverton, MD: Institut National de la Statistique and ORC Macro; ICF; 2006. Available from: http://dhsprogram.com/pubs/pdf/FR183/FR183.pdf.

[16] National Institute of Statistics (NISR) [Rwanda], Ministry of Health (MOH) [Rwanda], ICF International . Rwanda Demographic and Health Survey 2010. Calverton, MD: National Institute of Statistics, Ministry of Health, ICF International; 2012. Available from: http://dhsprogram.com/pubs/pdf/FR259/FR259.pdf.

[17] National Institute of Statistics [Rwanda], Ministry of Finance and Economic Planning [Rwanda], Ministry of Health [Rwanda], ICF International . Rwanda Demographic and Health Survey 2014–15. Kigali, Rwanda: National Institute of Statistics, Ministry of Finance and Economic Planning, Ministry of Health, and ICF International; 2016. Available from: http://dhsprogram.com/pubs/pdf/FR316/FR316.pdf.

[18] Rwanda Ministry of Health . Behavioral and Biological Surveillance Survey among female sex workers, Rwanda–2010. Survey report. Kigali; 2010.

[19] Rwanda Ministry of Health . National HIV and viral hepatitis annual report 2020–2021. 2021.

[20] World Bank . Population estimates and projections [Internet]. 2020. [cited 2025 Jan 24]. Available from: https://datacatalog.worldbank.org/dataset/population‐estimates‐and‐projections.

[21] Joint United Nations Programme on HIV/AIDS . Sex workers: population size estimate. UNdata; 2015. [updated 2024 July 3, cited 2015 October 21]. Available from: http://data.un.org/Data.aspx?d=UNAIDS&f=inID%3A111.

[22] Tuyishime E , Kayitesi C , Musengimana G , Malamba S , Moges H , Kankindi I , et al. Population size estimation of men who have sex with men in Rwanda: three‐source capture‐recapture method. JMIR Publ Health Surveill. 2023;9:e43114. 10.2196/43114 PMC10131990 36972131

[23] Twahirwa Rwema JO , Lyons CE , Herbst S , Liestman B , Nyombayire J , Ketende S , et al. HIV infection and engagement in HIV care cascade among men who have sex with men and transgender women in Kigali, Rwanda: a cross‐sectional study. J Int AIDS Soc. 2020;23:e25604. 33000912 10.1002/jia2.25604 PMC7527755

[24] Boily M‐C , Baggaley RF , Wang L , Masse B , White RG , Hayes RJ , et al. Heterosexual risk of HIV‐1 infection per sexual act: systematic review and meta‐analysis of observational studies. Lancet Infect Dis. 2009;9(2):118–129. 19179227 10.1016/S1473-3099(09)70021-0 PMC4467783

[25] Vitinghoff E , Douglas J , Judon F , McKiman D , MacQueen K , Buchinder SP . Per‐contact risk of human immunodeficiency virus transmission between male sexual partners. Am J Epidemiol. 1999;150(3):306–11. 10430236 10.1093/oxfordjournals.aje.a010003

[26] Weller SC , Davis‐Beaty K . Condom effectiveness in reducing heterosexual HIV transmission. Cochrane Database Syst Rev. 2002; (1):CD003255.11869658 10.1002/14651858.CD003255

[27] Supervie V , Viard JP , Costagliola D , Breban R . Heterosexual risk of HIV transmission per sexual act under combined antiretroviral therapy: systematic review and Bayesian modeling. Clin Infect Dis. 2014;59(1):115–122. 24723286 10.1093/cid/ciu223 PMC4305135

[28] Braunstein SL , Ingabire CM , Geubbels E , Vyankandondera J , Umulisa MM , Gahiro E , et al. High burden of prevalent and recently acquired HIV among female sex workers and female HIV voluntary testing center clients in Kigali, Rwanda. PLoS One. 2011;6(9):e24321. 21949704 10.1371/journal.pone.0024321 PMC3176231

[29] Binagwaho A , Chapman J , Koleros A , Utazirubanda Y , Pegurri E , Gahire R . Exploring HIV risk among MSM in Kigali, Rwanda. 2010.

[30] Braunstein SL , Umulisa MM , Veldhuijzen NJ , Kestelyn E , Ingabire CM , Nyinawabega J , et al. HIV diagnosis, linkage to HIV care, and HIV risk behaviors among newly diagnosed HIV‐positive female sex workers in Kigali, Rwanda. J Acquir Immune Defic Syndr. 2011;57(4):70–76. 10.1097/QAI.0b013e3182170fd3 21407083

[31] Nsanzimana S , Kanters S , Remera E , Forrest JI , Binagwaho A , Condo J , et al. HIV care continuum in Rwanda: a cross‐sectional analysis of the national programme. Lancet HIV. 2015;2(5):e208–e215. 26423003 10.1016/S2352-3018(15)00024-7

[32] Ntale RS , Rutayisire G , Mujyarugamba P , Shema E , Greatorex J , Frost SDW , et al. HIV seroprevalence, self‐reported STIs and associated risk factors among men who have sex with men: a cross‐sectional study in Rwanda, 2015. Sex Transm Infect. 2019;95(1):71–74. 29680827 10.1136/sextrans-2017-053311 PMC6580868

[33] Okal DO , Oyaro B , Zeh C , Desai MA , Samandari T , Chen RT , et al. Effect of point‐of‐care CD4 cell count results on linkage to care and antiretroviral initiation during a home‐based HIV testing campaign: a non‐blinded, cluster‐randomised trial. Lancet HIV. 2017;4(9):e393–e401. 28579225 10.1016/S2352-3018(17)30091-7

[34] Mocroft A , Phillips AN , Gatell J , Ledergerber B , Fisher M , Clumeck N , et al. Normalisation of CD4 counts in patients with HIV‐1 infection and maximum virological suppression who are taking combination antiretroviral therapy: an observational cohort study. Lancet. 2007;370(9585):407–413. 17659333 10.1016/S0140-6736(07)60948-9

[35] Losina E , Yazdanpanah Y , Deuffic‐Burban S , Wang B , Wolf LL , Messou E , et al. The independent effect of highly active antiretroviral therapy on severe opportunistic disease incidence and mortality in HIV‐infected adults in Côte d'Ivoire. Antiv Ther. 2007;12(4):543–551. PMC3073611 17668563

[36] Lankiewicz E , Sharp A , Drake P , Sherwood J , Macharia B , Ighodaro M , et al. Early impacts of the PEPFAR stop‐work order: a rapid assessment. J Int AIDS Soc. 2025;28(2):e26423. 39964153 10.1002/jia2.26423 PMC11834162

[37] The Foundation for AIDS Research . Impact of stop work orders for PEPFAR programs. 2025. accessed January 21, 2026. Available from: https://www.amfar.org/wp‐content/uploads/2025/01/Impact‐of‐Stop‐Work‐Orders‐for‐PEPFAR‐Programs‐2.pdf.

[38] World Health Organization . The impact of suspensions and reductions in health official development assistance on health systems. Rapid WHO country office stock take. Summary of results from 108 WHO country offices, 7 March–2 April 2025 (10 April 2025 version). 2025.

[39] O'Bryan GL . Factors associated with ART non‐adherence and contributing influence of stock shortages at Nkongsamba Regional Hospital, Cameroon. 2015.

[40] CNBC Africa . Inside Rwanda's HIV strategy, what's working & why it matters for Africa. 2025.

[41] Rwanda Ministry of Health . Rwanda HIV and AIDS national strategic plan. 2018. accessed January 21, 2026. Available from: https://hivpreventioncoalition.unaids.org/sites/default/files/attachments/rwanda_national_strategic_plan_on_hiv_and_aids_final.pdf.

[42] The U.S. President's Emergency Plan for AIDS Relief . 2021 Rwanda HIV/AIDS sustainability index and dashboard. 2021. accessed January 21, 2026 Available from: https://2021‐2025.state.gov/wp‐content/uploads/2022/06/Rwanda.pdf.

[43] Gandhi AR , Bekker L‐G , Paltiel AD , Hyle EP , Ciaranello AL , Pillay Y , et al. Potential clinical and economic impacts of cutbacks in the President's Emergency Plan for AIDS Relief program in South Africa: a modeling analysis. Ann Intern Med. 2025; ;178(4):457–467. 39932732 10.7326/ANNALS-24-01104 PMC11996594

[44] Ten Brink D, Martin‐Hughes R , Bowring AL , Wulan N , Burke K , Tidhar T , et al. Impact of an international HIV funding crisis on HIV infections and mortality in low‐income and middle‐income countries: a modelling study. Lancet HIV. 2025;12(5):e346–e354. 40157378 10.1016/S2352-3018(25)00074-8

[45] Tram KH , Ratevosian J , Beyrer C . By executive order: the likely deadly consequences associated with a 90‐day pause in PEPFAR funding. J Int AIDS Soc. 2025;28(3):e26431. 39996580 10.1002/jia2.26431 PMC11851316

[46] Joint United Nations Programme on HIV/AIDS . Impact of US funding cuts on the global AIDS response — Weekly update 10 March 2025. 2025. accessed January 21, 2026. Available from: https://www.unaids.org/en/resources/presscentre/featurestories/2025/march/20250312_sitrep.

[47] Rwanda Ministry of Health . National HIV Annual Report. 2014–2015. 2015.

[48] Rwanda Ministry of Health . National HIV and Viral Hepatitis Annual Report. 2019–2020. 2020.

[49] Republic of Rwanda . Country Progress Report. January 2008–December 2009. 2010.

[50] Rwanda Biomedical Center . Rwanda National HIV Annual Report. 2015–2016. 2016.

[51] Nsanzimana S , Mills EJ , Harari O , Mugwaneza P , Karita E , Uwizihiwe JP , et al. Prevalence and incidence of HIV among female sex workers and their clients: modelling the potential effects of intervention in Rwanda. BMJ Glob Health. 2020;5(8):e002300. 10.1136/bmjgh-2020-002300 PMC7412619 32764126

[52] Rwanda Biomedical Center (RBC) . Rwanda population‐based HIV impact assessment (RPHIA), 2018–2019. 2020.

[53] The World Bank . Population estimates and projections. 2020. accessed July 2, 2024. Available from: https://datacatalog.worldbank.org/dataset/population‐estimates‐and‐projections.

