# Supplementary material for: Sustaining Rwanda's HIV response after elimination of PEPFAR funding: a modelling analysis of HIV epidemic and care continuum outcomes
Source: J Int AIDS Soc. 2026 Feb 11;29(2):e70078. doi: 10.1002/jia2.70078 (PMC12894777; doi:10.1002/jia2.70078)
Supplement: Supplementary file 1 — Supporting Information [file JIA2-29-e70078-s001.pdf]

Supplementary Content

for

Sustaining Rwanda's HIV response after elimination of PEPFAR funding: a modeling analysis of HIV epidemic and care continuum outcomes

April D. Kimmel, Zhongzhe Pan, Gad Murenzi, Ellen Brazier, Batya Elul, Benjamin Muhoza, Marcel Yotebieng, Kathryn Anastos, Denis Nash, and on behalf of the Central Africa International epidemiology Databases to Evaluate AIDS (CA-IeDEA)

Supplementary Methods

Model description

We used the CA-IeDEA Rwanda HIV policy model, a deterministic dynamic model of the HIV epidemic among adults aged 15–64 years [1]. The model includes compartments for HIV disease progression by CD4 stratum and care engagement (i.e., undiagnosed, diagnosed, linked, on ART with suppressed HIV viral load, on ART but not suppressed, lost to follow-up (LTFU)). The modeled population is stratified into 35 sub-populations by age, sex, risk (including female sex workers (FSWs) and men who have sex with men (MSM)), and urbanicity to capture differences in HIV acquisition risk. The model captures transmission dynamics, including HIV treatment as prevention. The probability of HIV acquisition varies across susceptible sub-populations according to a sub-population's frequency of sexual contacts and probabilities of sexual contact with a given sub-population, condom use, and having a virally suppressed partner with HIV within the sub-population. Using the calibrated model [1], the model projects HIV epidemic and care continuum outcomes over ten years. Epidemic outcomes include: HIV prevalence (%), HIV incidence (number of new infections per 1,000 population), annual number of new HIV infections, number with HIV and cumulative HIV-related deaths. Care continuum outcomes include the percentage: diagnosed with HIV, on ART among those diagnosed (henceforth on ART among diagnosed), HIV virally suppressed among those on ART (henceforth, virally suppressed given ART), and on ART among those with HIV. We reported incremental differences and percentage

change in mean projected outcomes for *50% sustained, 75% sustained,* and *25% sustained* compared, separately, to *100% sustained* between 2025 and 2035.

Model inputs and calibration

As described previously [1], model inputs for the total population's initial distribution, including those living with and not living with HIV, were derived from individual-level longitudinal data from the Rwanda cohort of CA-IeDEA [2], estimated from national surveys and population projections [3-9], and extracted from the literature [10-12] (**Table 1**, main text). Sub-population-specific model parameters for susceptible population growth and HIV acquisition came from adult population projections and the literature [3-8, 13-18]. Transition probabilities for natural history disease progression and movement along the HIV care continuum were derived from the Rwanda cohort of CA-IeDEA [2], estimated from national surveys, and extracted from the literature [3-5, 19-22]. We assigned distributions to model inputs, randomly drew input values from each distribution, generated sets of randomly selected inputs, and projected historical model outcomes, 2005–2020. Model projections for each set of randomly selected inputs were compared to 47 calibration targets reflecting epidemiologic, global goals and care engagement indicators, 2005–2020. A goodness-of-fit measure informed selection of the top 50 best-fitting sets of model inputs that were used in the current analysis. Approximately 80% of projections were a good or acceptable fit to calibration targets, suggesting projections fit historical data and the model can be used to inform policy.

Supplementary References

1. Kimmel AD, Pan Z, Brazier E, Murenzi G, Mujwara D, Muhoza B, et al. Development and calibration of a mathematical model of HIV outcomes among Rwandan adults: Informing achievement of global targets across sub-populations in Rwanda. PLoS One. 2025;20(5):e0310662. PubMed PMID: 40367028. PMCID: PMC12077668.
2. National Institute of Allergy and Infectious Diseases. Central Africa International epidemiology Databases to Evaluate AIDS. National Institute of Allergy and Infectious Diseases. Available from: https://ca-iedea.org/.
3. Institut National de la Statistique (INSR) [Rwanda], ORC Macro. Rwanda Demographic and Health Survey 2005 [Dataset]. Calverton, Maryland, USA: Institut National de la Statistique and ORC Macro [Producers]; ICF [Distributor], 2006. Available from: http://dhsprogram.com/pubs/pdf/FR183/FR183.pdf.
4. National Institute of Statistics (NISR) [Rwanda], Ministry of Health (MOH) [Rwanda], ICF International. Rwanda Demographic and Health Survey 2010 [Dataset]. Calverton, Maryland, USA: National Institute of Statistics, Ministry of Health, ICF International [Producers]; ICF [Distributor], 2012. Available from: http://dhsprogram.com/pubs/pdf/FR259/FR259.pdf.
5. National Institute of Statistics [Rwanda], Ministry of Finance and Economic Planning[Rwanda], Ministry of Health[Rwanda], ICF International. Rwanda Demographic and Health Survey 2014-15 [Dataset]. Kigali, Rwanda: National Institute of Statistics, Ministry of Finance and Economic Planning, Ministry of Health, and ICF International [Producers]; ICF [Distributor], 2016. Available from: http://dhsprogram.com/pubs/pdf/FR316/FR316.pdf.
6. Rwanda Ministry of Health. Behavioral and biological surveillance survey among female sex workers, Rwanda – 2010: Survey report. 2010. Available from: https://rbc.gov.rw/IMG/pdf/behavioral_and_biological_survey_among_female_sex_workers_rwanda_2010.pdf.
7. Rwanda Ministry of Health. National HIV and viral hepatitis annual report 2020-2021. 2021. Available from: https://www.rbc.gov.rw/index.php?id=693.
8. World Bank. Population estimates and projections. Available from: https://datacatalog.worldbank.org/dataset/population-estimates-and-projections.

9. Joint United Nations Programme on HIV/AIDS. Sex workers: Population size estimate. UNdata; 2015. [updated 2024 July 3. 2015 October 21. Available from: http://data.un.org/Data.aspx?d=UNAIDS&f=inID%3A111.
10. The U.S. President's Emergency Plan for AIDS Relief. Rwanda country operational plan (COP/ROP) 2023 strategic direction summary 2022. Available from: https://www.prepwatch.org/wp-content/uploads/2024/06/Rwanda-Strategic-Direction-Summary-2023.pdf.
11. Tuyishime E, Kayitesi C, Musengimana G, Malamba S, Moges H, Kankindi I, et al. Population size estimation of men who have sex with men in Rwanda: Three-source capture-recapture method. JMIR Publ Health Surveill. 2023;9:e43114.
12. Twahirwa Rwema JO, Lyons CE, Herbst S, Liestman B, Nyombayire J, Ketende S, et al. HIV infection and engagement in HIV care cascade among men who have sex with men and transgender women in Kigali, Rwanda: a cross-sectional study. J Int AIDS Soc. 2020;23:e25604.
13. Boily M-C, Baggaley RF, Wang L, Masse B, White RG, Hayes RJ, et al. Heterosexual risk of HIV-1 infection per sexual act: systematic review and meta-analysis of observational studies. Lancet Infect Dis. 2009;9(2):118-29.
14. Vitinghoff E, Douglas J, Judon F, McKiman D, MacQueen K, Buchinder SP. Per-contact risk of human immunodificiency virus tramnsmision between male sexual partners. Am J Epidemiol. 1999;150(3):306-11.
15. Weller SC, Davis-Beaty K, . Condom effectiveness in reducing heterosexual HIV transmission. Cochrane Database Syst Rev. 2002 (1):CD003255.
16. Supervie V, Viard JP, Costagliola D, Breban R. Heterosexual risk of HIV transmission per sexual act under combined antiretroviral therapy: Systematic review and Bayesian modeling. Clin Infect Dis. 2014;59(1):115-22.
17. Braunstein SL, Ingabire CM, Geubbels E, Vyankandondera J, Umulisa MM, Gahiro E, et al. High burden of prevalent and recently acquired HIV among female sex workers and female HIV voluntary testing center clients in Kigali, Rwanda. PLoS One. 2011;6(9):e24321. PubMed PMID: 21949704.
18. Binagwaho A, Chapman J, Koleros A, Utazirubanda Y, Pegurri E, Gahire R. Exploring HIV Risk among MSM in Kigali, Rwanda. 2010.

19. Braunstein SL, Umulisa MM, Veldhuijzen NJ, Kestelyn E, Ingabire CM, Nyinawabega J, et al. HIV diagnosis, linkage to HIV Care, and HIV risk behaviors among newly diagnosed HIV-positive female sex workers in Kigali, Rwanda. J Acquir Immune Defic Syndr. 2011;57(4):70-6.
20. Nsanzimana S, Kanters S, Remera E, Forrest JI, Binagwaho A, Condo J, et al. HIV care continuum in Rwanda: A cross-sectional analysis of the national programme. Lancet HIV. 2015;2(5):e208-e15.
21. Ntale RS, Rutayisire G, Mujyarugamba P, Shema E, Greatorex J, Frost SDW, et al. HIV seroprevalence, self-reported STIs and associated risk factors among men who have sex with men: a cross-sectional study in Rwanda, 2015. Sex Transm Infect. 2019;95(1):71-4.
22. Okal DO, Oyaro B, Zeh C, Desai MA, Samandari T, Chen RT, et al. Effect of point-of-care CD4 cell count results on linkage to care and antiretroviral initiation during a home-based HIV testing campaign: a non-blinded, cluster-randomised trial. Lancet HIV. 2017;4(9):e393-e401.
